# Noble-metal-free Co_3_S_4_–S/G porous hybrids as an efficient electrocatalyst for oxygen reduction reaction†

**DOI:** 10.1039/c6sc00357e

**Published:** 2016-03-02

**Authors:** Wenling Gu, Liuyong Hu, Wei Hong, Xiaofang Jia, Jing Li, Erkang Wang

**Affiliations:** a State Key Laboratory of Electroanalytical Chemistry, Changchun Institute of Applied Chemistry, Chinese Academy of Sciences Changchun Jilin 130022 PR China ekwang@ciac.ac.cn; b State Key Laboratory of Polymer Physics and Chemistry, Changchun Institute of Applied Chemistry, Chinese Academy of Sciences Changchun Jilin 130022 PR China; c University of the Chinese Academy of Sciences Beijing 100049 PR China

## Abstract

Developing of a new noble-metal-free catalyst to replace Pt-based catalysts of the oxygen reduction reaction (ORR) both in alkaline and acidic conditions is extremely significant for the fuel cell. In this paper, based on the pyrolysis of an inexpensive precursor cobalt dithiolene (a S_4_-chelate complex) on simultaneously reduced graphene oxide (GO) as a support matrix, a high-efficiency noble-metal-free hybrid for oxygen reduction reaction (ORR) consisting of Co_3_S_4_ nanoparticles encapsulated in porous sulfur doped graphene (referred as Co_3_S_4_–S/G) was fabricated. The catalyst obtained at 800 °C (Co_3_S_4_–S/G-800) manifests excellent oxygen reduction activity. Of note, the Co_3_S_4_–S/G-800 hybrids also exhibited prominent ORR activity with high selectivity (mainly 4e^−^ reaction process) and very low H_2_O_2_ yield in acidic electrolyte. The optimal Co_3_S_4_–S/G-800 hybrid displayed much greater tolerance to methanol and higher stability than that of Pt/C. These admirable performances endorse Co_3_S_4_–S/G-800 electrocatalyst holding great potential for fuel cells. Meanwhile, this work also provides a simple and practical method to fabricate cobalt chalcogenides by using the cost-effective and easily synthesized S_4_-chelate complex.

## Abstract 2

A high-efficiency noble-metal-free oxygen reduction reaction (ORR) catalyst was obtained based on the pyrolysis of the inexpensive precursor cobalt dithiolene (an S_4_-chelate complex) on simultaneously reduced graphene oxide (GO) as a support matrix.

## Graphical abstract

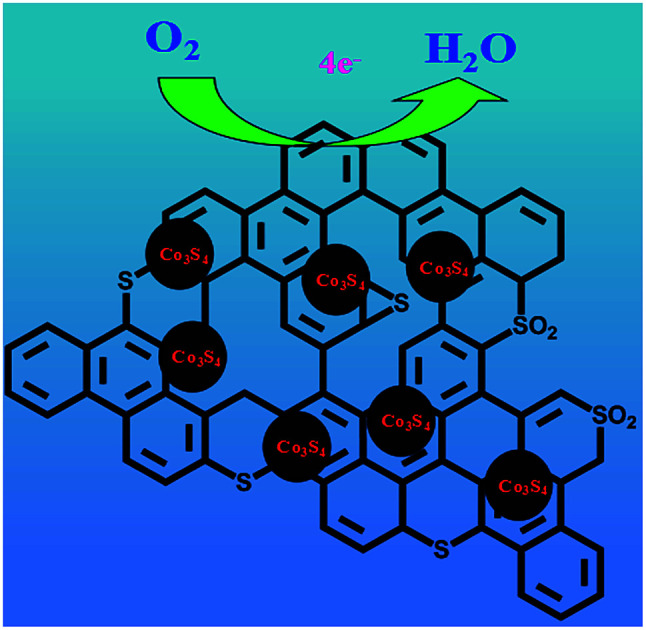

## Introduction

With the rapid development of technology energy demand grows continuously. As a high energy-conversion efficiency technique, fuel cells have attracted much attention in recent years.^1^ The oxygen reduction reaction (ORR) is a crucial step in high energy-conversion devices. However, the sluggish cathodic reaction of ORR often needs the assistance of an efficient electrocatalyst.^2^ Among these electrocatalysts, Pt and Pt-based alloy materials^3–5^ have exhibited remarkable performance for ORR. Nevertheless, their high price, their scarcity, and especially low stability for methanol crossover have limited their widespread use.^6^ Correspondingly, much effort has been made to design and synthesize non-precious metal electrocatalysts (NPMCs) as alternatives to Pt, such as heteroatom-doped carbon materials, metal–N_*x*_ macrocycles, metal oxides supported on graphene, metal chalcogenides *etc.*^7–10^ Since Jasinski discovered that cobalt phthalocyanine (an N_4_-chelate macrocycle) possessed good ORR activity in alkaline conditions,^11^ metal–N_4_ macrocycles based catalysts (M–N_4_/C) have received much attention owing to the highly active site of surface nitrogen associated with metal.^12–20^ Müllen *et al.*^13^ made another attempt to obtain a [CoN_4_]_3_/C electrocatalyst by using a new class of metal–N_4_ macrocyclic complexes as a precursor, which showed high catalyst activity and durability for ORR in alkaline conditions. Niu's group^14^ had reported that by pyrolyzing a mixture of cyanocobalamin (an N_4_-chelate macrocycle, vitamin B12) and GO, an excellent electrocatalyst was fabricated with a positive onset potential and high durability for ORR in alkaline electrolyte. Although excellent activity of M–N_4_/C based catalysts has been obtained in alkaline conditions, only few of these catalysts were found to retain catalyst activity in acidic conditions, mostly owing to the low amounts of catalytic sites of these catalysts. Moreover, these macrocycles still suffer from high-price and complicated synthetic processes which significantly influences their practical application.

In this work, inspired by the excellent electrocatalytic activity of M–N_4_/C catalysts and the special structure of the N_4_-chelate macrocycles, we proposed to use the cost-effective and easily fabricated metal–S_4_ complex of cobalt dithiolene as a cobalt and sulfur rich precursor for obtaining high-performance ORR catalysts for the first time. By facile pyrolysis of the cobalt dithiolene and GO (which acts as a support carbon matrix) at 800 °C, a non-precious metal electrocatalyst of Co_3_S_4_ nanoparticles encapsulated in porous sulfur-doped graphene (Co_3_S_4_–S/G-800) was obtained. As far as we know, cobalt chalcogenides (such as Co_1−*x*_S, CoS, Co_3_S_4_, Co_9_S_8_) had been shown to display higher chemical stability, electrical conductivity and electrocatalytic activity than other metal chalcogenides.^21,22^ With such outstanding advantages, they have been extensively applied in many fields, such as optoelectronic devices, energy storage, magnetic devices and electrocatalysis. In addition, theoretical studies also predicted that cobalt chalcogenides have great potential in ORR.^23^ However, most recently reported cobalt chalcogenide based ORR catalysts required complicated and time-consuming synthesis processes and showed far lower electrocatalytic activity than the commercial Pt/C catalyst. Herein, the obtained Co_3_S_4_–S/G-800 hybrid was found to be a highly effective and robust catalyst to boost ORR. Co_3_S_4_–S/G-800 also showed better electrochemical durability and tolerance toward methanol than commercial Pt/C. To our surprise, the ORR activity of Co_3_S_4_–S/G-800 was superior to most recently reported cobalt sulfide nanoparticles or other heteroatom based electrocatalysts both in alkaline and acidic conditions.^21–28^ All these results demonstrate that the obtained Co_3_S_4_–S/G-800 is a promising electrocatalyst for fuel cells and that the pure S_4_-chelate complex can be used to replace the high-price N_4_-chelate macrocycles (such as cyanocobalamin, phthalocyanine, porphyrins *etc.*) for designing high-performance ORR catalysts. Meanwhile, this work also provides a simple and practical method to fabricate cobalt chalcogenides.

## Results and discussion

**Fig. 1:**
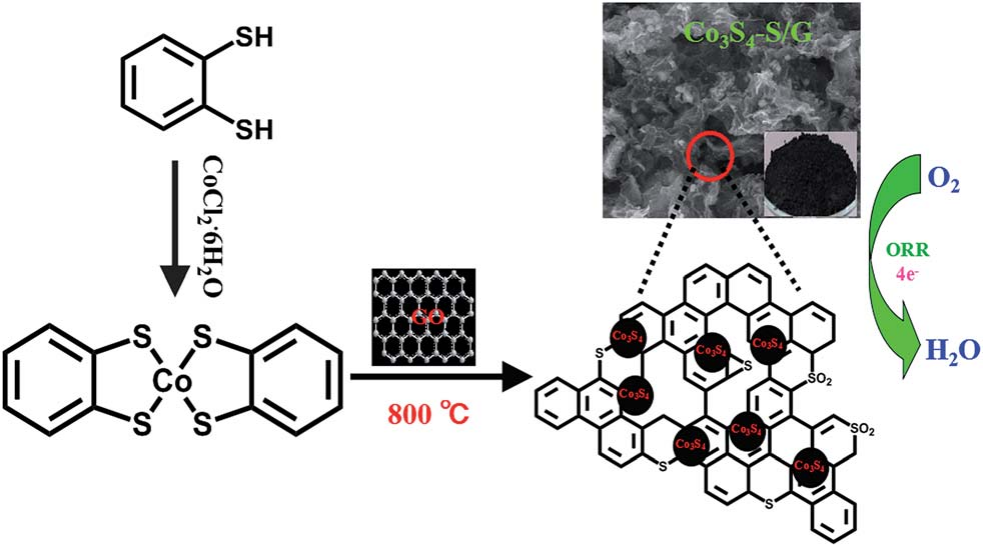
Schematic illustration of the fabrication process of Co_3_S_4_–S/G catalysts and electrocatalytic activity for oxygen reduction reaction.

Fig. 1 depicts the chemical structure of the cobalt dithiolene (which can be easily obtained by a one-step process), the synthetic process of the Co_3_S_4_–S/G and the corresponding photograph of the catalyst. It is well known that the carbonization temperature is a key factor to influence the performance of the catalysts on ORR, since a low pyrolysis temperature will facilitate the incorporation of activated elements into the carbon skeleton and a high temperature may effectively increase electrical conductivity of the catalysts.^29^ Therefore, the catalysts were obtained at different pyrolysis temperatures to find the optimum pyrolysis condition. XRD was carried out to analyze the crystal structures of all the as-prepared catalysts at different temperatures. As shown in Fig. S3a,† the presence of a peak around 25° suggested that the ordered graphitic phase was formed at high carbonization temperatures, which was beneficial for the electrical conductivity of the catalysts. Meanwhile, the obtained catalysts showed the typical crystal structure of Co_3_S_4_ with the peaks located at 18.38, 30.01, 35.6, 46.6, 52.3 and 54.4°, which correspond to the (111), (220), (311), (400), (511) and (440) planes (JCPDS file: #471738) respectively.

The properties of the derived catalysts were further analyzed by Raman spectra. From Fig. S3b,† two broad bands corresponding to the D-band (∼1350 cm^−1^) and G-band (∼1580 cm^−1^) were observed. The D-band is related to the vibrations of the sp^3^ carbon atoms of disordered graphene nanosheets, while the G-band is attributed to the in-plane vibrations of sp^2^ carbon atoms of graphite. The ratio of the D band to the G band (*I*_D_/*I*_G_) varied with the pyrolysis temperatures, with values of 0.967 (600 °C), 0.985 (700 °C), 1.00 (800 °C) and 1.03 (900 °C). The increased ratio of *I*_D_/*I*_G_ proved that the disordered and significant edge sites had been successfully increased by raising the pyrolysis temperature which would effectively enhance the conductivity of the catalysts and help charge localization for O_2_ chemisorption.^30^

The morphology of the fabricated Co_3_S_4_–S/G catalysts at different pyrolysis temperatures was studied using SEM. As observed in Fig. S4,† raising pyrolysis temperatures from 600 to 900 °C, the Co_3_S_4_ nanoparticles were gradually generated and uniformly encased in the derived carbon skeleton. The elemental compositions of prepared Co_3_S_4_–S/G catalysts were investigated by XPS and EDX. From Table S1,† the contents of cobalt and sulfur showed decreased trends with increasing pyrolysis temperatures. It is of note that the contents of cobalt and sulfur were found to be lower in the XPS data compared with EDX, which may be caused by the generated carbon layers around the Co_3_S_4_ nanoparticles at a higher pyrolysis temperature and the impermeability to carbon layers to XPS for analyzing the cobalt and sulfur elements. However, this phenomenon provided favorable stability to the catalyst in catalytic aspects, especially under some strict conditions. Based on previous reports, although the encapsulated Co_3_S_4_ nanoparticles in the carbon layers could not directly contact with the electrolyte, they could activate the outer carbon layers making them more active toward ORR.^31^

Moreover, in order to explore the influence of the as-obtained catalysts at different temperatures on ORR, the electrochemical experiments were first evaluated by CV and RRDE techniques in 0.1 M KOH (Fig. S5 and 6†) electrolyte. It should be noticed that by comparing the onset potential, half-wave potential as well as current density, the Co_3_S_4_–S/G-800 exhibited superior catalytic activity than the samples prepared at other temperatures (Table S2†), implying that the catalyst obtained at 800 °C showed the optimal synergetic effect between the excellent electrical conductivity of the support matrix and the added component. Fig. S7 and Table S3† display the Brunauer–Emmet–Teller (BET) surface areas of all the catalysts, and the Co_3_S_4_–S/G-800 showed the largest surface area of 52.44 m^2^ g^−1^. In addition, based on the Barrett–Joyner–Halenda (BJH) analysis, the Co_3_S_4_–S/G-800 catalyst contained mesopores with a peak at 13 nm, which may exert essential transport ability for ORR relevant substances (O_2_, H^+^/OH^−^, H_2_O) and provide more active sites.^32^ It is believed that the relatively larger surface area and existing mesopore feature were both conducive to ORR of Co_3_S_4_–S/G-800 catalyst. Therefore, the Co_3_S_4_–S/G catalyst discussed below refers to this sample unless otherwise specified.

**Fig. 2:**
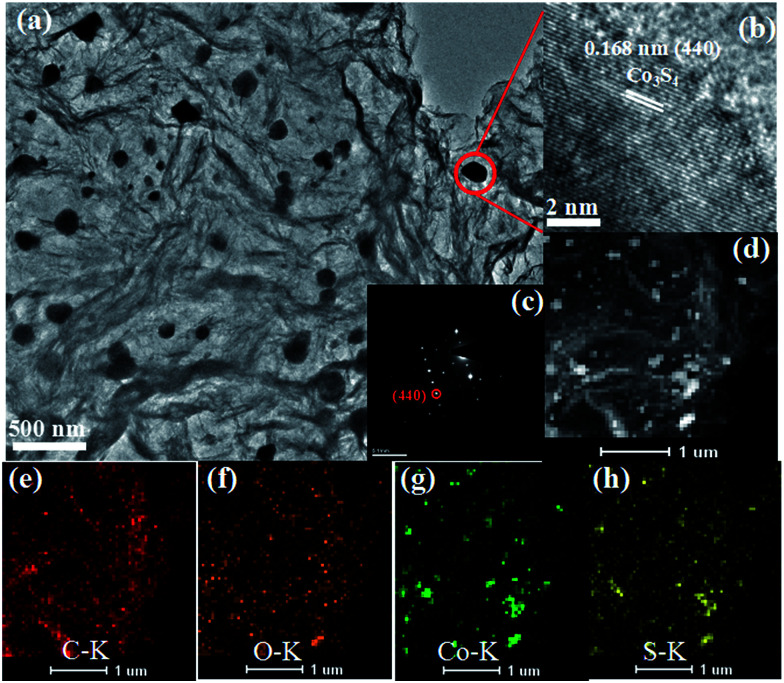
TEM images (a), HRTEM image (b), SAED pattern (c), HAADF-STEM (d) and elemental mapping images (e–h) of Co_3_S_4_–S/G-800 catalyst.

The TEM image of the Co_3_S_4_–S/G-800 (Fig. 2a) showed that the Co_3_S_4_ nanoparticles are uniformly distributed in the graphene matrix. Moreover, the HRTEM image (Fig. 2b) and SAED pattern (Fig. 2c) were both investigated to better understand the nanoparticles. The lattice distance of 0.168 nm should correspond to the (440) crystal planes of the Co_3_S_4_ phase. Fig. 2d–h show the HAADF-STEM and elemental mapping images of the catalyst. It could be confirmed that the Co_3_S_4_ nanoparticles were grown in the graphene matrix and the sulfur element was present not only in Co_3_S_4_ nanoparticles, but was also distributed over the support graphene matrix. It is proposed that the Co_3_S_4_ nanoparticles originate from the decomposition of cobalt dithiolene at high temperature. Moreover, a part of cobalt dithiolene served as the source of sulfur during the pyrolysis process.

**Fig. 3:**
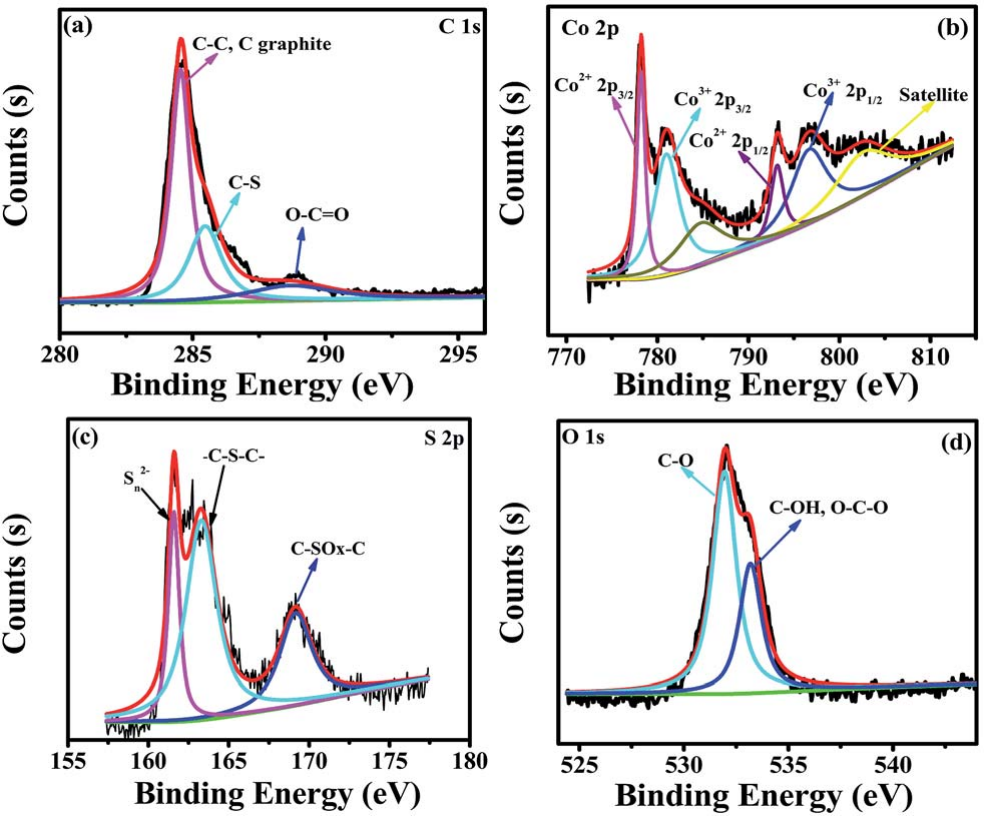
High-resolution C 1s XPS spectra (a), Co 2p spectra (b), S 2p spectra (c) and O 1s spectra (d) of the as-obtained Co_3_S_4_–S/G-800 hybrids.

The elemental composition of Co_3_S_4_–S/G-800 was measured using EDX analysis. As shown in Fig. S8,† the four elements C, O, Co, S were observed. XPS measurements were used to confirm the definite chemical state of the four elements in the Co_3_S_4_–S/G-800. Fig. 3a shows the high-resolution C 1s spectra with the peak located at 286.4 eV which should correspond to the C–S group, along with C–C (291.87 eV) and O–C=O (284.11 eV) groups, indicating the major sp^2^ carbon atom environments of Co_3_S_4_–S/G-800.^33^ The Co 2p spectrum shown in Fig. 3b could be divided into six peaks which were assigned to 2p_3/2_ of Co^2+^ and Co^3+^ ions, 2p_1/2_ of Co^2+^ and Co^3+^ ions, as well as the corresponding satellite peaks, which indicated the presence of Co_3_S_4_ nanoparticles. Based on previous reports, the peaks in the S 2p spectrum in Fig. 3c at 161.85 and 163.58 eV were assigned to S_*n*_^2−^ and –C–S–C– , respectively,^34^ while the peak at 169.62 eV was from C–SO_*x*_ groups. In order to confirm the S signals in the Co_3_S_4_–S/G-800 were due to covalent C–S bonds, and did not arise from physically absorbed S, the Co_3_S_4_–S/G-800 sample was washed ultrasonically with deionized water or alcohol and remeasured. As expected, the XPS spectra did not exhibit any difference, thus proving the S is doped in the graphene matrix. In addition, from the Raman spectra of the pristine graphene in Fig. S9,† it could be observed the G band occurred at ∼1589 cm^−1^, while under the same conditions, the G-band for Co_3_S_4_–S/G-800 sample appeared at ∼1580 cm^−1^ (Fig. S3b†). According to the report of Zhu and co-workers,^35^ it is believed that the matrix of Co_3_S_4_–S/G-800 may show n-type doping of graphene. This characteristic, coupled with the XPS results, could strongly certify that the S atoms were doped in the graphene of Co_3_S_4_–S/G-800 hybrids *via* covalent bonds *via* C–S–C bonds. Indeed, owing to the larger atomic radius of S (103 pm) than C (77 pm), the S doped mesopore catalyst will provide more favorable strains and defects for ORR. Fig. 3d shows the small quantity of oxygen in the Co_3_S_4_–S/G-800 hybrids, the residual oxygen may be caused by the incomplete reduction of GO.

**Fig. 4:**
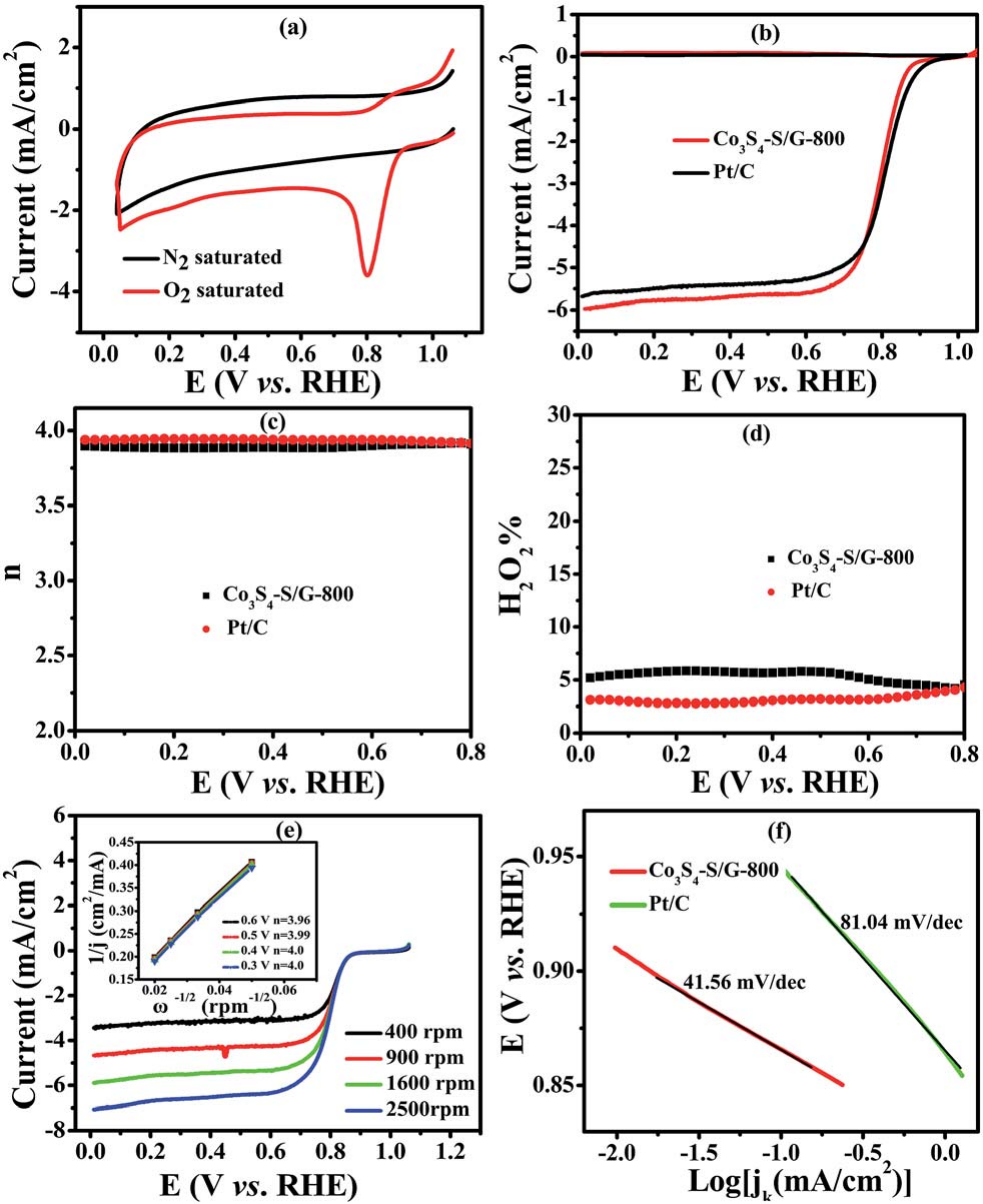
CVs (a) of the Co_3_S_4_–S/G-800 nanocatalyst modified electrode in N_2_ or O_2_ saturated 0.1 M KOH electrolyte with a potential scan rate of 50 mV s^−1^; RRDE voltammograms (b), electron transfer number (c), H_2_O_2_ yield (d) of the Co_3_S_4_–S/G-800 and Pt/C catalysts in O_2_ saturated 0.1 M KOH electrolyte at a scan rate of 5 mV s^−1^. The rotation rate is 1600 rpm; RDE voltammograms (e) of the Co_3_S_4_–S/G-800 at various rotation rates and the Koutecky–Levich plots (shown as inset). The corresponding Tafel plots (f) of the Co_3_S_4_–S/G-800 and Pt/C catalysts.

To compare the catalyst behavior of Co_3_S_4_–S/G-800 relative to commercial Pt/C, typical CV experiments were first carried out in N_2_ or O_2_ saturated electrolyte with a potential scan rate of 50 mV s^−1^. As shown in Fig. 4a, a large cathodic ORR peak of Co_3_S_4_–S/G-800 in O_2_ saturated 0.1 M KOH electrolyte could be easily observed with the onset potential of 0.92 V. In addition, the RRDE voltammograms were further carried out to investigate the ORR catalytic activity of these catalysts. We could see that the Co_3_S_4_–S/G-800 expressed nearly identical catalyst activity to Pt/C (Fig. 4b). In general, during the reduction of oxygen, a complete four electron (4e^−^) reaction process was regarded as more favorable than a two electron (2e^−^) process, owing to the low hydrogen peroxide yield (H_2_O_2_%). Generally, the electrode transfer number and H_2_O_2_% were calculated from the tested disk current and ring current. The result (Fig. 4d) showed that the H_2_O_2_% of Co_3_S_4_–S/G-800 was less than 6% in the potential range from 0.8 to 0 V in the alkaline electrolyte, indicating an almost four electron (4e^−^) reaction process as found for Pt/C (*n* ≈ 4.0, Fig. 4c). To further explore the ORR mechanism of the Co_3_S_4_–S/G-800 catalyst, RDE measurements were performed. According to the Koutecky–Levich equation, plots of *J*^−1^*vs. ω*^−1/2^ at different electrode potentials were obtained (the inset of Fig. 4e) and the electrode transfer number can be calculated from the slope of these lines. Values were calculated to be 3.96, 3.99, 4.0 and 4.0 at the potentials of 0.6, 0.5, 0.4 and 0.3 V, respectively, in 0.1 M KOH. These results were in accordance with the RRDE technique, showing a 4e^−^ reaction process for ORR. Fig. 4f shows the Tafel plots of Co_3_S_4_–S/G-800 and Pt/C derived from Fig. 4b. Co_3_S_4_–S/G-800 has a Tafel slope of 41.56 mV per decade in 0.1 M KOH. In fact, many transition-metal based ORR catalysts after a pyrolysis process possess similar Tafel slopes. Such a Tafel slope proved that the ORR rate determining step should be the splitting of the O–O bands when two electrons moved from active sites to the chemisorbed O_2_ molecules. Therefore, these values proved that the Co_3_S_4_–S/G-800 catalyst had excellent kinetic characteristics for the reduction of oxygen.

**Fig. 5:**
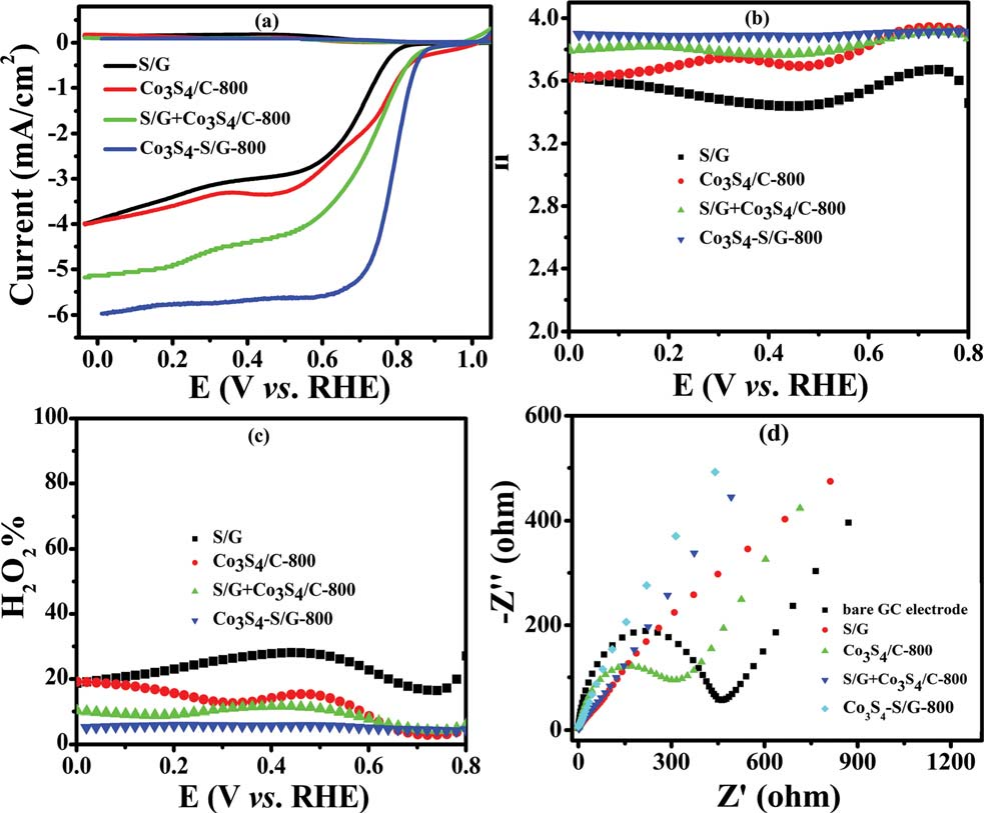
LSV curves (a) electron transfer number (b) and H_2_O_2_ yield (c) of S/G, Co_3_S_4_/C-800, S/G + Co_3_S_4_/C-800 (physical mixture) and Co_3_S_4_–S/G-800 catalysts in an O_2_-saturated 0.1 M KOH electrolyte with a scan rate of 5 mV s^−1^. EIS (d) of S/G, Co_3_S_4_/C-800, S/G + Co_3_S_4_/C-800 and Co_3_S_4_–S/G-800 modified working electrode in a solution of 5.0 mM Fe(CN)_6_^3−/4−^ containing 100 mM KCl.

In contrast, the comparative catalysts of S/G, Co_3_S_4_/C-800 (Fig. S10a and b† show the XRD pattern and TEM image) and S/G + Co_3_S_4_/C-800 (physical mixture) showed a lower electron transfer number of 3.43–3.62 for S/G, 3.62–3.86 for Co_3_S_4_/C-800 and 3.8–3.88 for S/G + Co_3_S_4_/C-800 (Fig. 5b), indicating inferior electrocatalysis selectivity and electron transfer ability for these comparative samples. Fig. 5d shows the EIS plots of the comparative catalyst modified electrodes. It was well-known that the semicircle regions at high-ac modulation frequency of the Nyquist plot is related to the electrode transfer process, while the line regions at low-ac modulation frequency represent the diffusion process. Compared with the simple combinations or physical mixture, the Co_3_S_4_–S/G-800 catalyst modified electrode exhibited an almost straight line at high-frequency regions, demonstrating the high electric conduction ability of the catalyst and the strong coupling between Co_3_S_4_ and S/G, which significantly affected the electronic structure of the support graphene matrix.

Moreover, Co_3_S_4_–S/G-800 also displayed excellent electrocatalytic activity in acidic solution. As shown in Fig. S11b,† the Co_3_S_4_–S/G-800 catalyst exhibited an onset potential of 0.80 V in 0.5 M H_2_SO_4_ electrolyte. Most important, the electron transfer number of the Co_3_S_4_–S/G-800 was calculated as close to four electrons (Fig. S11c†) in 0.5 M H_2_SO_4_ and the H_2_O_2_ yield is very low under the investigated potential (Fig. S11d†). Hence, the low H_2_O_2_ yield and high electrode transfer number in both alkaline and acidic conditions clearly indicate that Co_3_S_4_–S/G-800 possesses remarkable ORR catalytic efficiency. As far as we know, few reports on cobalt sulfide nanoparticles based electrocatalysts have shown higher ORR performance compared with the Co_3_S_4_–S/G-800 in both alkaline and acidic conditions (Table S4†).

**Fig. 6:**
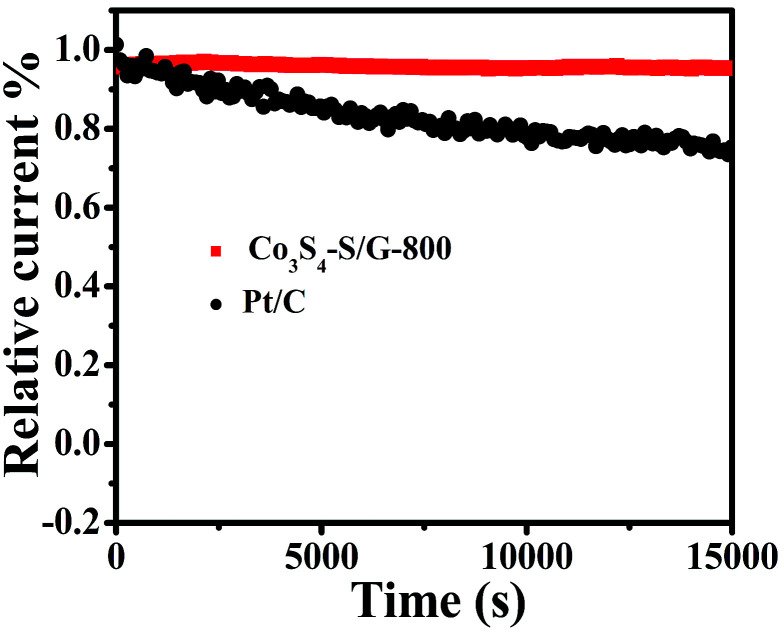
Amperometric *i*–*t* curves of Co_3_S_4_–S/G-800 and Pt/C in O_2_-saturated 0.1 M KOH electrolyte.

For commercialization, the durability and tolerance toward methanol is another important aspect for a fuel cell. As shown in Fig. S12 and 13,† almost no change of the LSV or CV curves was observed for Co_3_S_4_–S/G-800 both in alkaline and acidic conditions, indicating little effect of methanol on the catalyst. In contrast, an obvious change of the onset potential, half-wave potential and the ORR peak can be found for the Pt/C catalyst. These phenomena reveal that the Co_3_S_4_–S/G-800 catalyst was superior to the commercial Pt/C for methanol fuel cells. Since poor tolerance is a major obstacle of non-noble metal catalysts for fuel cells, especially in harsh acidic conditions,^36^ it is essential to measure the stability of ORR catalysts. Herein, amperometric *i*–*t* tests were carried out to measure the stability of the Co_3_S_4_–S/G-800 with a long time of 15 000 s. Impressively, as shown in Fig. 6 and S14,† after 15 000 s, the Co_3_S_4_–S/G-800 exhibited a relatively slower decay than the commercial Pt/C in both alkaline and acidic conditions. Moreover, by comparing recently reported cobalt sulfide nanoparticle based electrocatalysts, such as Co_1−*x*_S/RGO hybrid,^24^ CoS_2_-based thin films^25^ and CoS_2_/N, S-GO,^26^ the Co_3_S_4_–S/G-800 also showed a higher thermal stability. This result convincingly exemplifies that the Co_3_S_4_–S/G-800 catalyst has favorable stability. The fabricated Co_3_S_4_–S/G-800 catalyst with these excellent features may thus hold a promising potential for fuel cells.

Therefore, based on the characterization of the Co_3_S_4_–S/G-800 catalyst, the displayed prominent ORR activity could be attributed to these effects, (1) the high electric conduction of the support matrix; (2) the S-doped catalyst provides more favorable strains and defects for ORR; (3) the mesoporous structure in the Co_3_S_4_–S/G-800 exerts good transport ability for ORR relevant substances (O_2_, H^+^/OH^−^, H_2_O) and provides more exposed active sites; (4) a synergistic effect between the S-doped graphene and Co_3_S_4_ nanoparticles.

## Conclusions

In summary, we successfully prepared a metal–S_4_ complex to replace traditional high-price N_4_-chelate macrocycles. From this a high-efficiency noble-metal-free catalyst Co_3_S_4_–S/G-800 was synthesized on a large scale by a simple and facile annealing of the inexpensive precursor and GO at 800 °C. The as-prepared Co_3_S_4_–S/G-800 catalyst showed excellent ORR catalytic activity which was comparable with commercial Pt/C. Moreover, as a noble metal-free catalyst, the Co_3_S_4_–S/G-800 manifested obviously low methanol crossover effects, and robust stability in a long time test experiment. All these results demonstrate that the S_4_-chelate complex can serve as an effective precursor for designing the ORR catalyst, which opens up a new strategy to achieve highly efficient, stable, and low-cost ORR catalysts. Moreover, based on this work, an easy, economical and practical method to fabricate cobalt chalcogenides also was provided by using the pure S_4_-chelate complex as an efficient precursor.

## Experimental

### Chemicals and reagents

Graphite powder (spectral pure) was from Alfa Aesar (Ward Hill, MA, USA). Absolute ethanol, methanol, sulfuric acid (H_2_SO_4_) and potassium hydroxide (KOH) were purchased from Beijing Chemical Reagent (Beijing, China). 1,2-Benzenedithiol (TCI) and 20% E-TEK Pt/C were obtained from Alfar Aesar (Tianjin, China) and Millipore Mill-Q (18.2 MΩ cm) deionized water was used to prepare the aqueous solutions.

### Apparatus

Transmission electron microscopy (TEM) images, high angle annular dark field-scanning transmission electron microscopy (HAADF-STEM) and elemental mapping images were recorded by a TECNAI G2 high-resolution transmission electron microscope (Hitachi, Tokyo, Japan) with an accelerating voltage of 200 kV. Energy dispersive X-ray (EDX) spectra and scanning electron microscope (SEM) images were measured with an XL30 ESEM FEG SEM (Philips, Netherlands) operating with an accelerating voltage of 20 kV. X-Ray photoelectron spectroscopy (XPS) analysis was obtained from an ESCALAB-MKII X-ray photoelectron spectroscope (VG Scientific, UK). Powder X-ray diffraction (XRD) was recorded by a D8 ADVANCE (Germany) using Cu-Kα radiation (*λ* = 1.5406 Å). Raman spectra were recorded by a Renishaw 2000 model confocal microscopy Raman spectrometer (Renishaw Ltd., Gloucestershire, UK). Electrochemical impedance spectroscopy (EIS) was measured with an Autolab/PG30 electrochemical analyzer system (ECO Chemie B.V. Netherlands).

In addition, cyclic voltammetric (CV) and amperometric *i*–*t* curves were carried out with a CH Instruments 800 voltammetric analyzer (Shanghai, China). Rotating ring-disk electrode (RRDE) and rotating disk electrode (RDE) measurements were made using a Model RRDE-3A Apparatus (ALS, Japan) coupled with a CH Instruments 800 electrochemical workstation. In the electrochemical experiments, the modified glassy carbon electrode with catalyst samples acted as the working electrode, with an Ag/AgCl (saturated KCl) electrode and a platinum wire as the reference electrode and counter electrode, respectively. All electrode potentials were referenced to the reversible hydrogen electrode (RHE) using the formula *E* (*vs.* RHE) = *E* (*vs.* Ag/AgCl) + 0.197 + 0.059pH. In 0.1 M KOH solution (pH = 13), *E* (*vs.* RHE) = *E* (*vs.* Ag/AgCl) + 0.964, while, in 0.5 M H_2_SO_4_ electrolyte (pH = 0.25), *E* (*vs.* RHE) = *E* (*vs.* Ag/AgCl) + 0.212.

### Preparation of the catalyst samples

As illustrated in Fig. S1,† the S_4_-chelate complex of cobalt dithiolene was obtained by a previously reported method in a one-step process,^37^ and the UV-Vis absorption spectrum (Fig. S2†) was used to characterize the compound. Then, in a typical synthesis of Co_3_S_4_–S/G-800 samples, 2 g cobalt dithiolene was dissolved into 100 mL absolute ethanol containing 2 mg mL^−1^ GO (which was obtained by a modified Hummers' procedure^38^) with ultrasonication and stirring until a homogeneous solution was obtained. Afterwards, the solvent was removed under reduced pressure, and the remaining powder was thermally annealed under flowing Ar at 180 and 800 °C for 1 and 2 h, respectively, with a heating rate of 5 °C min^−1^. After that, the obtained black products were etched in 50 mL of 0.5 M H_2_SO_4_ solution for 24 h to remove unstable and inactive substance and then were washed three times with deionized water. The Co_3_S_4_–S/G-800 catalyst was obtained by drying the black product at 60 °C in a vacuum.

The comparative samples of pristine graphene, sulfur-doped graphene (S/G, pyrolysis of 1,2-benzenedithiol and GO at 800 °C), Co_3_S_4_/C-800 (pyrolysis of cobalt dithiolene complex without GO), S/G + Co_3_S_4_/C-800 (physical mixture) and the nanocatalysts which were carbonized at 600, 700 and 900 °C (designated as Co_3_S_4_–S/G-600, Co_3_S_4_–S/G-700 and Co_3_S_4_–S/G-900, respectively) were also prepared to better understand the catalytic properties for ORR.

### Electrocatalytic activity measurements

Prior to each experiment, the working electrode was polished with 0.3 and 0.05 μm alumina slurries and cleaned with deionized water and absolute ethanol to obtain a mirror finish. The catalyst ink was prepared as follows: 4 mg catalyst sample was added into 1 mL of a mixed solution which contained 20 : 1 : 0.075 (v/v/v) of water, absolute ethanol and Nafion (5.0 wt%). Then the solution was sonicated for 30 min to obtain 4 mg mL^−1^ catalyst. Before the experiments of CVs and amperometric *i*–*t*, 6 μL catalyst ink was dropped onto a glassy carbon electrode (GCE: 3.0 mm in diameter) with a loading amount of 0.34 mg cm^−2^ and dried under an infrared lamp. The linear sweep voltammetry (LSV) measurements were recorded by using RRDE or RDE techniques to evaluate the electrocatalytic activity of different catalyst samples. In addition, the 20% E-TEK Pt/C catalyst was also prepared in the same way and dropped with a loading amount of 25 μg Pt cm^−2^ as a comparison.

Before each experiment, the electrolyte was bubbled by purging high-purity N_2_ gas for at least 30 min in order to remove dissolved oxygen. The modified working electrode was electrochemically treated and cleaned by CV sweeping (potential scan from 1.1 to 0 V (*vs.* RHE)) with a scan rate of 100 mV s^−1^ in an N_2_-saturated electrolyte until a reproducible curve was achieved. Meanwhile, CV curves in N_2_-saturated or O_2_-saturated solution were obtained by CV sweeping with a scan rate of 50 mV s^−1^ after purging N_2_ or O_2_ at least for 30 min. The amperometric *i*–*t* curves were obtained by sweeping the Co_3_S_4_–S/G-800 or 20% E-TEK Pt/C (Pt/C) catalyst modified electrode over 15 000 s at a potential of 0.614 V (*vs.* RHE) in O_2_-saturated 0.1 M KOH and 0.5 M H_2_SO_4_ solution.

Furthermore, in the RRDE experiments, the Pt ring potential was set at 1.264 V (*vs.* RHE) in 0.1 M KOH and 1.012 V (*vs.* RHE) in 0.5 M H_2_SO_4_. The transferred electron number (*n*) and the generated H_2_O_2_ can be calculated from the values of *i*_d_ (the current of disk electrode) and *i*_r_ (the current of ring current) using the following equations:^39^1
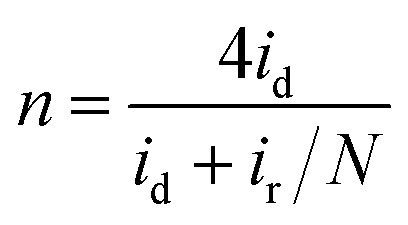
2
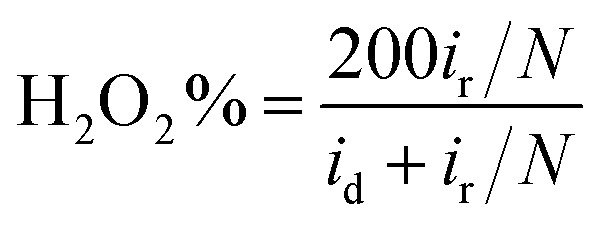
where *N* is the collection efficiency of the ring electrode (0.42).

The RDE measurements were performed in O_2_-saturated 0.1 M KOH or 0.5 M H_2_SO_4_ electrolyte by a negative-direction sweeping potential with a scan rate of 5 mV s^−1^ under different electrode rotation rates. The electron transfer also can be estimated according to the Koutecky–Levich equations:3
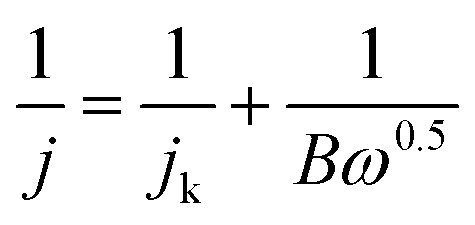

4 *B* = 0.2*nF*(*D*_0_)^2/3^*v*^−1/6^*C*_0_

where *j* represents the current density, *j*_k_ is the kinetic-limiting current density, *ω* is the electrode rotation rate, *n* is the transferred electron number, *F* is the Faraday constant of 96 485 C mol^−1^, and *D*_0_ is the diffusion coefficient of O_2_ (1.9 × 10^−5^ cm^2^ s^−1^ in 0.1 M KOH and 1.4 × 10^−5^ cm^2^ s^−1^ in 0.5 M H_2_SO_4_), *v* represents the kinematic viscosity of the electrolyte (0.01 cm^2^ s^−1^ both in 0.1 M KOH and 0.5 M H_2_SO_4_), *C*_0_ is the bulk concentration of O_2_ (1.2 × 10^−6^ mol cm^−3^ in 0.1 M KOH and 1.1 × 10^−6^ mol cm^−3^ in 0.5 M H_2_SO_4_).^40,41^

## Supplementary Material

SC-007-C6SC00357E-s001
