# Supplementary material for: Noble-metal-free Co3S4–S/G porous hybrids as an efficient electrocatalyst for oxygen reduction reaction
Source: Chem Sci. 2016 Mar 2;7(7):4167–73. doi: 10.1039/c6sc00357e (PMC6014111; doi:10.1039/c6sc00357e)
Supplement: SC-007-C6SC00357E-s001 [file SC-007-C6SC00357E-s001.pdf]

Electronic Supplementary Material (ESI) for Chemical Science.
This journal is © The Royal Society of Chemistry 2016

Supporting Information

Noble-Metal-Free Co_3S_4-S/G Porous Hybrids As an Efficient Electrocatalyst for Oxygen Reduction Reaction

Wenling Gu,[ac] Liuyong Hu,[bc] Wei Hong,[ac] Xiaofang Jia,[a] Jing Li*[a] and Erkang Wang*

[a]State Key Laboratory of Electroanalytical Chemistry, Changchun Institute of Applied Chemistry, Chinese Academy of Sciences, Changchun, Jilin 130022, PR China;

[b]State Key Laboratory of Polymer Physics and Chemistry, Changchun Institute of Applied Chemistry, Chinese Academy of Sciences, Changchun, Jilin 130022, PR China;

[c]University of the Chinese Academy of Sciences, Beijing, 100049, PR China

Corresponding author: Prof. Erkang Wang, Associate Prof. Jing Li, Tel: +86-431-85262003, Fax: +86-431-85689711, Email: ekwang@ciac.ac.cn and lijingce@ciac.ac.cn

Table of content

Figure S1	The fabrication process of the cobalt dithiolene.
Figure S2	The UV-Vis absorption spectra of the obtained cobalt dithiolene.
Figure S3	XRD patterns and Raman spectra of the Co_3S_4-S/G catalysts at different carbonization temperature.
Figure S4	SEM images of the catalysts which achieved at different instant carbonation temperature
Figure S5	CVs, LSV curves, electron transfer number and H_2O_2 yield of different catalysts in an O_2-saturated 0.1 M KOH electrolyte.
Figure S6	Tafel slopes of the different catalysts were obtained in alkaline conditions.
Figure S7	N_2 adsorption-desorption isotherm and BJH pore distributions of the obtained Co_3S_4-S/G catalyst.
Figure S8	EDX image of the obtained Co_3S_4-S/G-800 catalyst.
Figure S9	Raman spectra of pristine graphene.
Figure S10	XRD pattern and TEM image of the reference sample Co_3S_4/C-800.
Figure S11	CVs, RRDE voltammograms, electron transfer number, H_2O_2 yield and RDE voltammograms of Co_3S_4-S/G-800 hybrids in 0.5 M H_2SO_4 electrolyte.
Figure S12 & Figure S13	LSV & CVs of Co-S/C-800 and Pt/C in an O_2-saturated 0.1 M KOH and 0.5 M H_2SO_4 solution without and with 0.3 M CH_3OH.

Figure S14	The amperometric i-t curves of the Co_3S_4-S/G-800 and Pt/C in an O_2 saturated 0.5 M H_2SO_4 electrolyte.
Table S1	The Elemental compositions of different catalysts samples determined by XPS and EDX.
Table S2	Comparison of the Brunauer-Emmet-Teller (BET) surface area of the obtained catalysts.
Table S3	Comparison of the ORR ability of various catalysts.

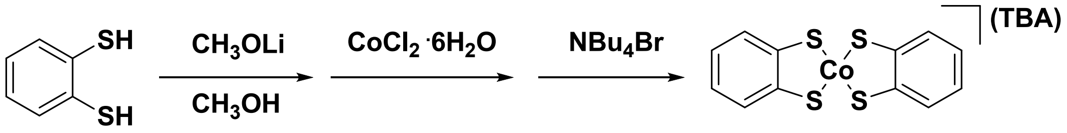

Figure S1 The fabrication process of the cobalt dithiolene.

1,2-benzedithiol (0.5 mmol) was added to a solution of CH_3OLi (2 mmol) in 5ml degassed methanol and stirred for 45 min. A solution of $Co(_6H_2O)Cl_2$ (0.3 mmol) was added in one portion, forming a blue solution. This mixture was stirred for 15 min, air was bubbled through the solution for 20 min, and then Bu_4NBr (0.6 mmol) added. The precipitate was filtered and washed with H_2O, methanol, and Et_2O.

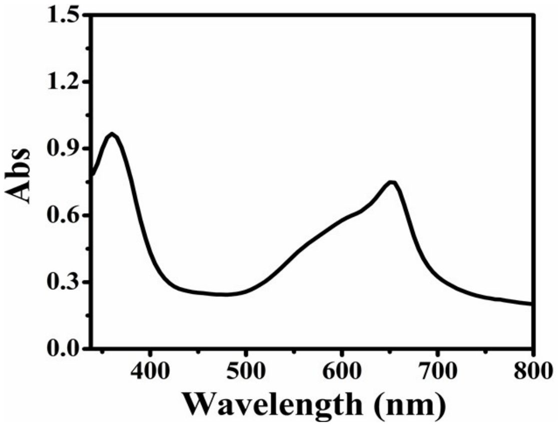

Figure S2 The UV-Vis absorption spectra of the obtained cobalt dithiolene.

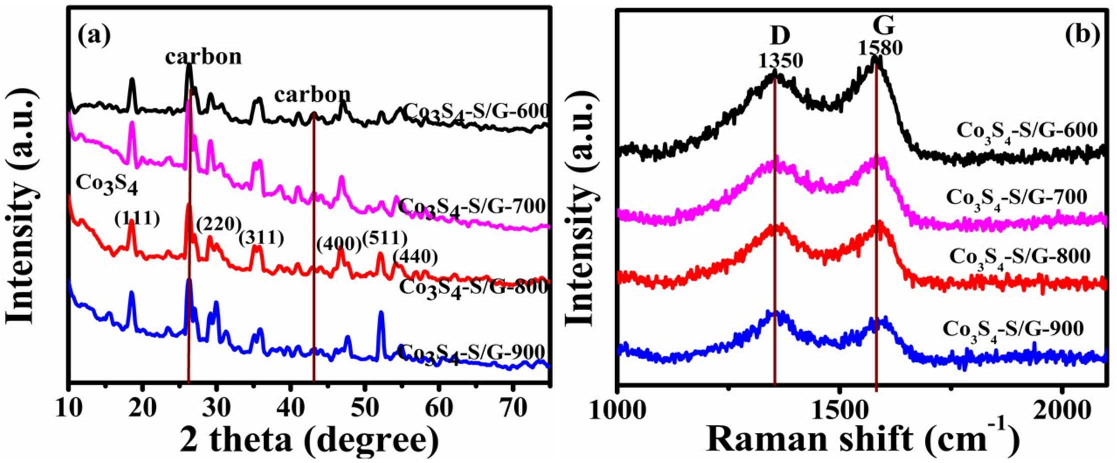

Figure S3 XRD patterns (a) and Raman spectra (b) of the Co_3S_4-S/G catalysts at different carbonization temperature from 600 °C to 900 °C.

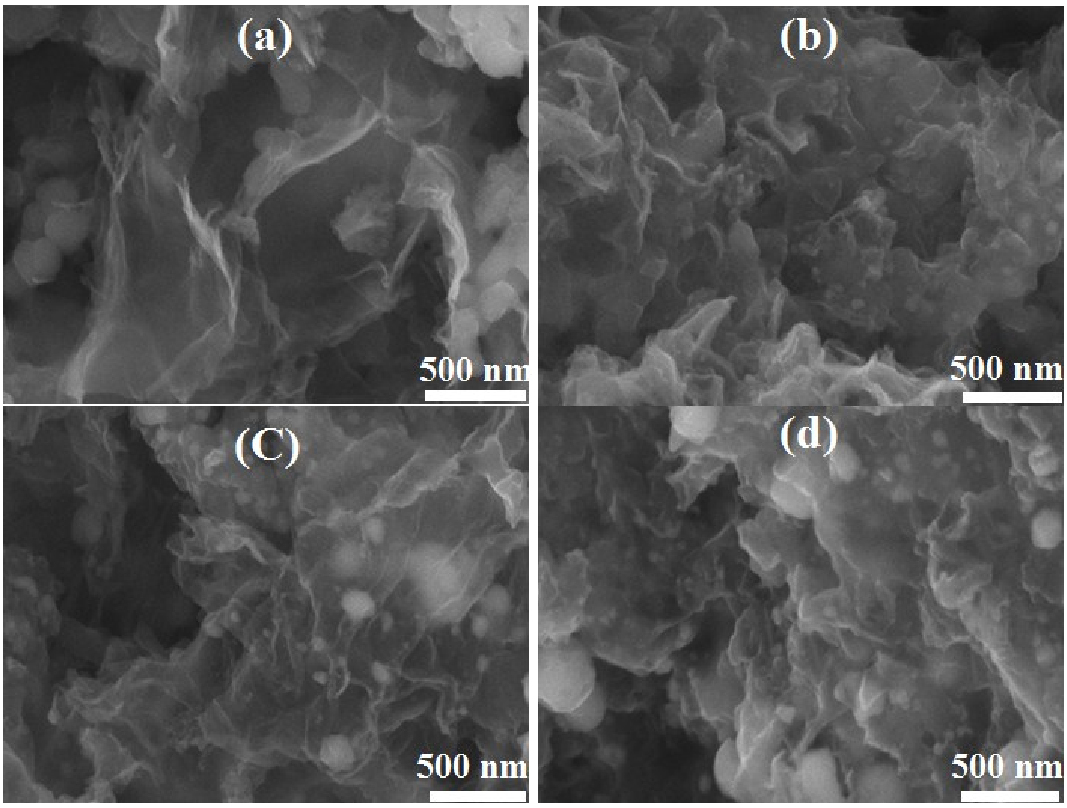

Figure S4 SEM images of the catalysts achieved at different instant carbonation temperature, (a) Co_3S_4-S/G-600, (b) Co_3S_4-S/G-700, (c) Co_3S_4-S/G-800, (d) Co_3S_4-S/G-900.

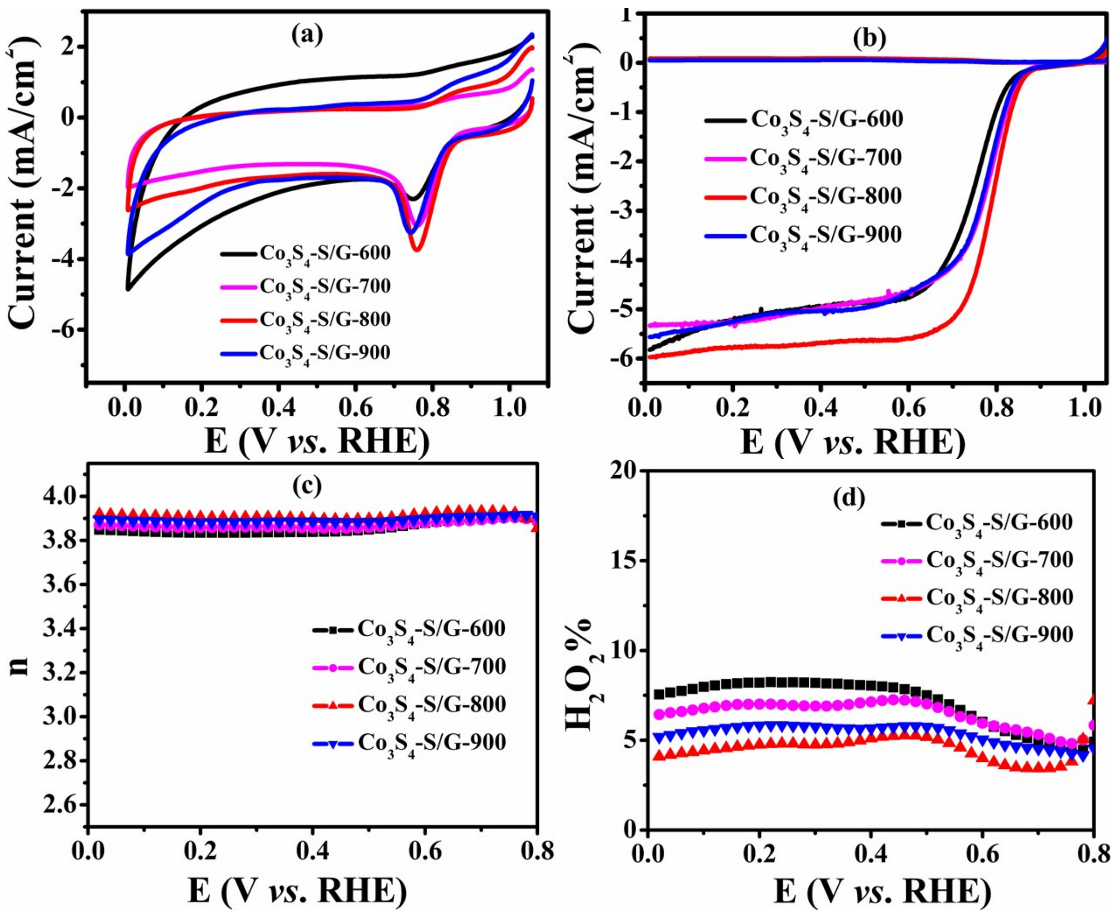

Figure S5 (a) CVs of the different catalysts were obtained in an O_2-saturated 0.1 M KOH electrolyte. The potential scan rate: 50 mV s^{-1}; (b) LSV curves (c) electron transfer number and (d) H_2O_2 yield of different nanocatalysts in an O_2-saturated 0.1 M KOH electrolyte with the scan rate of 5 mV/s. Rotation rate is 1600 rpm.

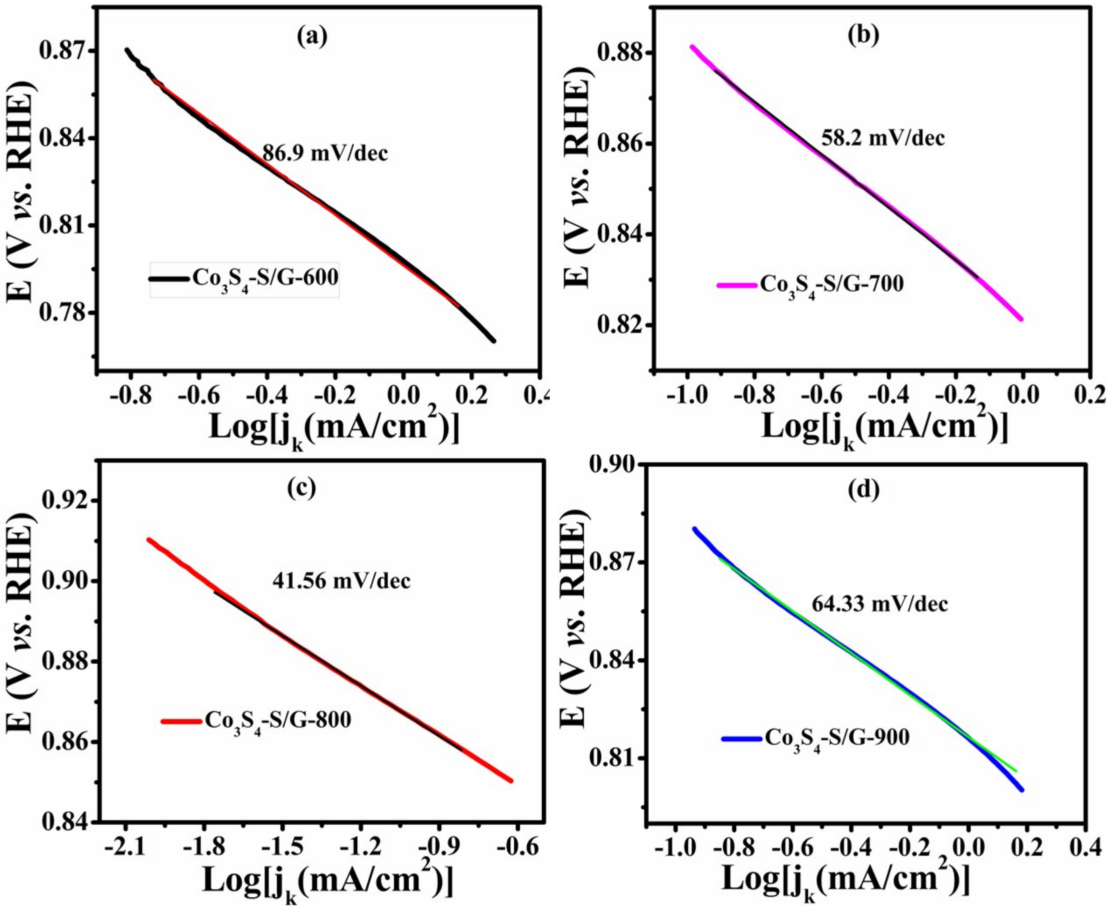

Figure S6 Tafel slopes of the different catalysts were obtained in alkaline conditions.

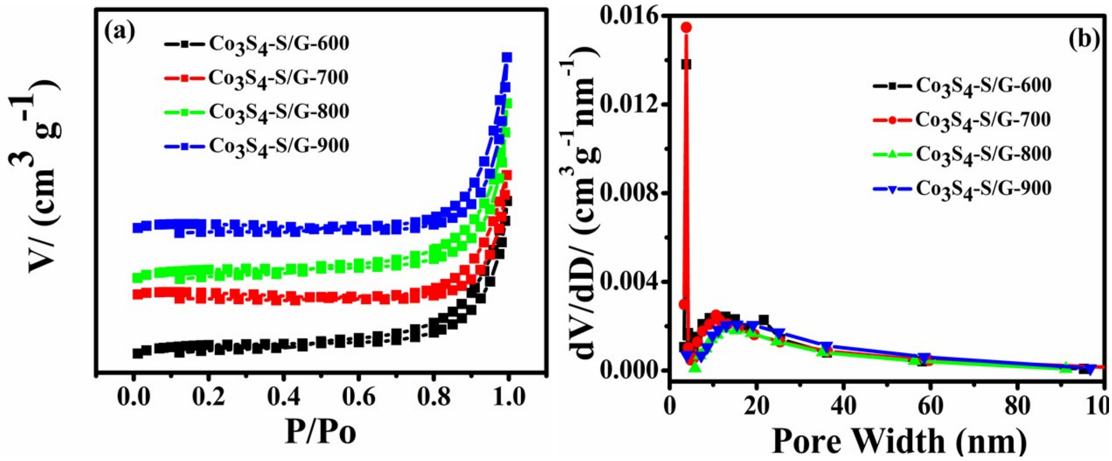

Figure S7 (a) N_2 adsorption-desorption isotherm and (b) BJH pore distributions of the obtained Co_3S_4-S/G catalysts.

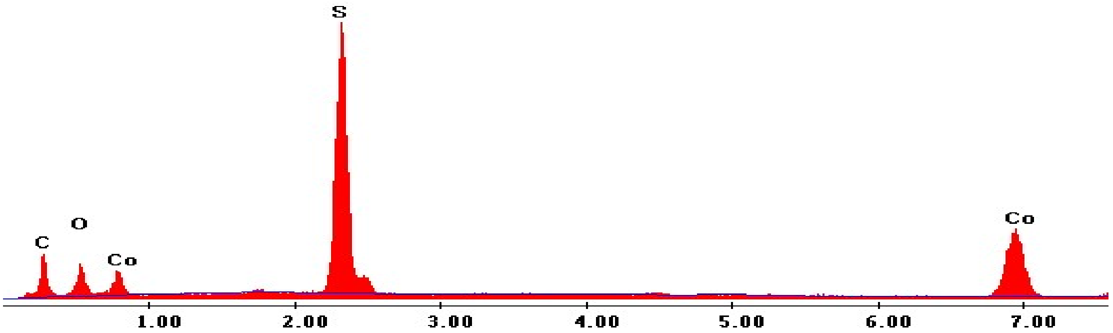

Figure S8 EDX image of the obtained Co_3S_4-S/G-800 catalyst.

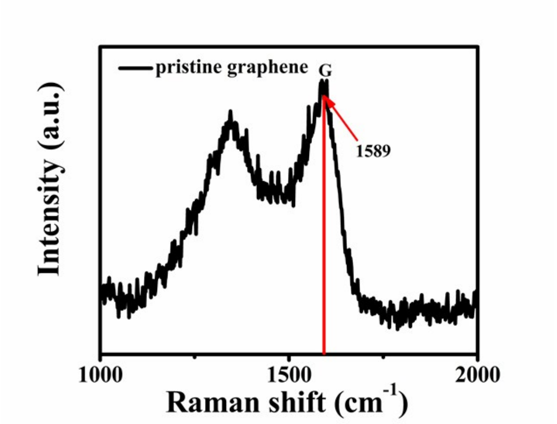

Figure S9 Raman spectra of pristine graphene.

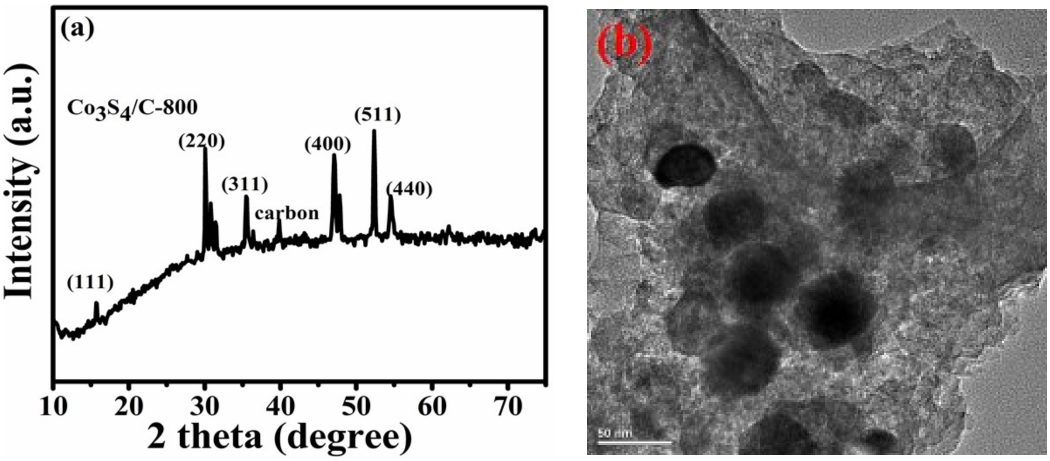

Figure S10 XRD pattern (a), TEM image (b) of the reference sample Co_3S_4/C-800.

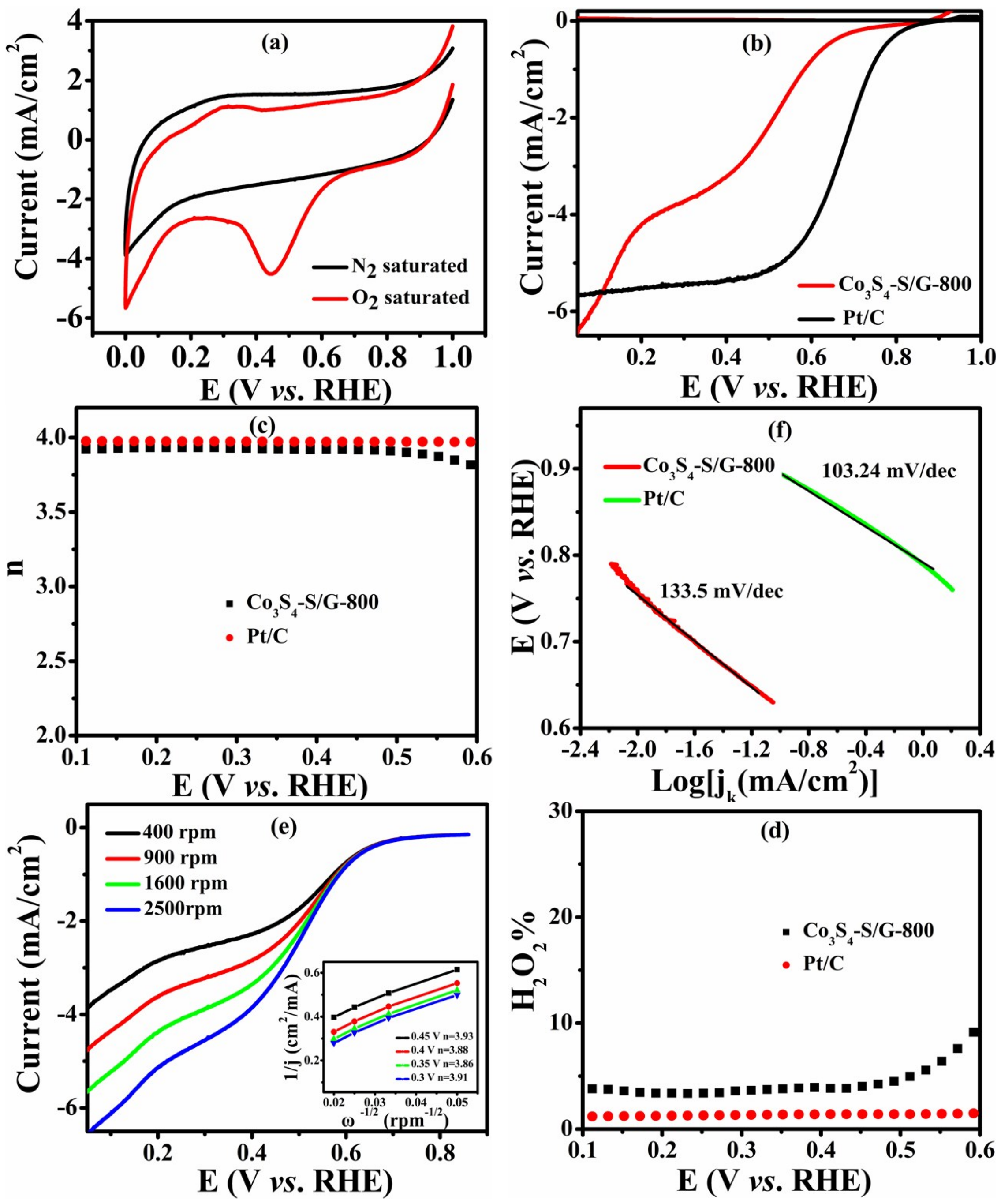

Figure S11 CVs (a) of the Co_3S_4-S/G-800 nanocatalyst in N_2 or O_2 saturated 0.5 M H_2SO_4 electrolyte at the potential scan rate of 50 mV s^{-1}; RRDE voltammograms (b), electron transfer number (c), H_2O_2 yield (d) of the Co_3S_4-S/G-800, Pt/C and Co_3S_4/C-800 hybrids in O_2 saturated 0.5 M H_2SO_4 electrolyte with a scan rate of 5 mVs^{-1}. The rotation rate was 1600 rpm; RDE voltammograms (e) of the Co_3S_4-S/G-800 at various rotation rates and the inset is the koutecky–levich plots for ORR using the Co_3S_4-S/G-800 hybrids at different potentials in 0.5 M H_2SO_4 electrolyte; The corresponding tafel plots (f) of the Co_3S_4-S/G-800 and Pt/C catalysts.

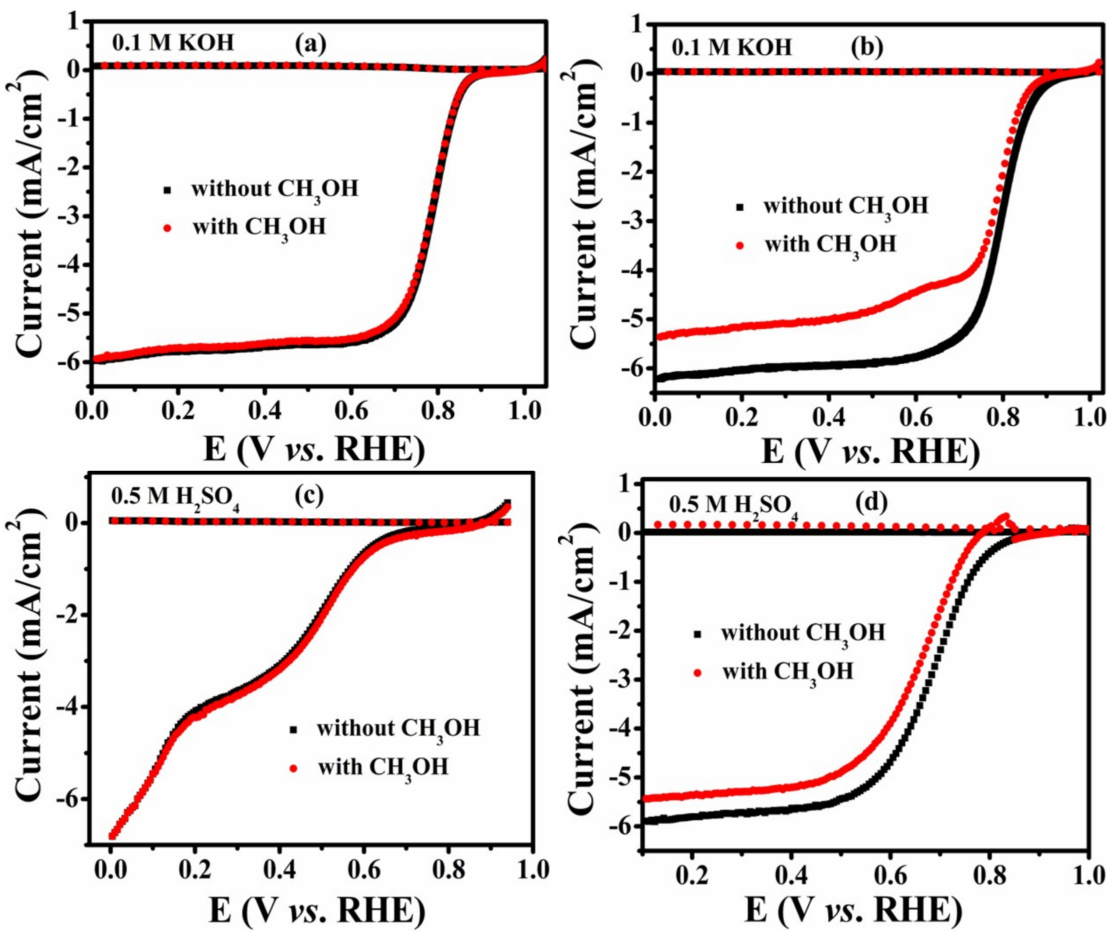

Figure S12 LSV curves of Co_3S_4-S/G-800 (a, c) and Pt/C (b, d) in an O_2-saturated 0.1 M KOH and 0.5 M H_2SO_4 solution without and with 0.3 M CH_3OH, the scan rate is 5 mVs^{-1}. Rotation rate is 1600 rpm.

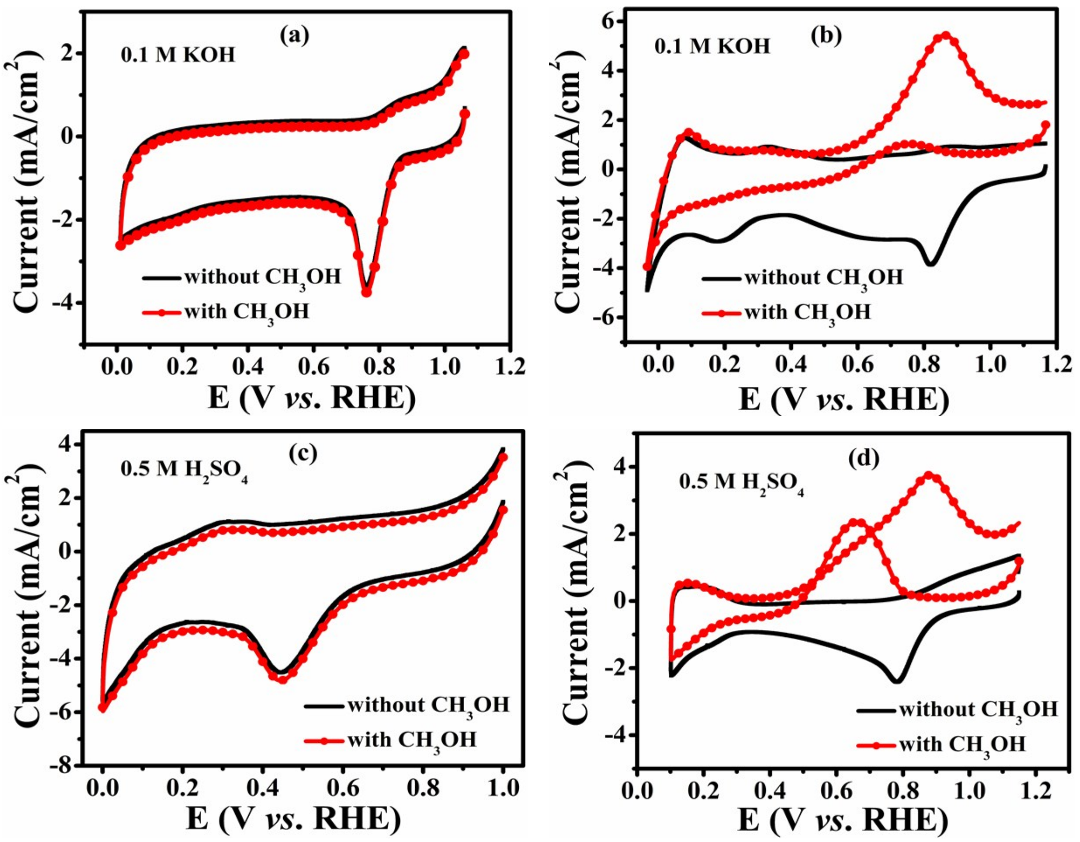

Figure S13 CVs of Co_3S_4-S/G-800 (a, c) and Pt/C (b, d) in an O_2-saturated 0.1 M KOH or 0.5 M H_2SO_4 solution without and with 0.3 M CH_3OH, the scan rate is 50 mV s^{-1}.

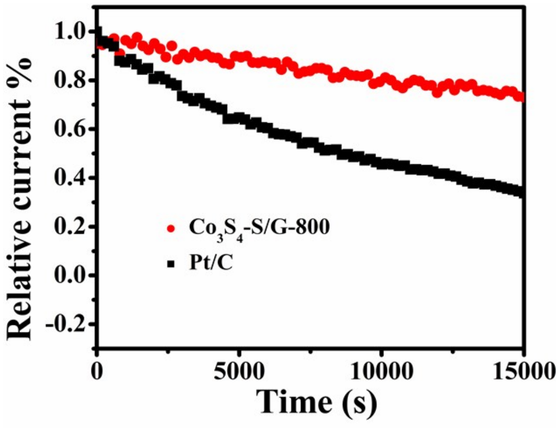

Figure S14 The amperometric i-t curves of the Co_3S_4-S/G-800 and Pt/C in an O_2 saturated 0.5 M H_2SO_4 electrolyte.

Table S1 The Elemental compositions of different catalysts samples determined by XPS and EDX.

Samples	elements compositions (%) determined by XPS/EDX	
	S atom %	Co atom %
Co_3S_4-S/G-600	2.21/11.42	1.7/8.29
Co_3S_4-S/G-700	2.08/11.32	1.64/8.1
Co_3S_4-S/G-800	2.38/10.9	1.35/7.59
Co_3S_4-S/G-900	1.56/9.30	1.3/7.52

Table S2 The catalyst activity of the catalysts at different carbonization temperature.

Samples	onset potential (V) vs. RHE	half-wave potential (V) vs. RHE	current density (mA/cm^2)
Co_3S_4-S/G-600	0.87	0.75	5.66
Co_3S_4-S/G-700	0.88	0.78	5.5
Co_3S_4-S/G-800	0.92	0.80	6.0
Co_3S_4-S/G-900	0.89	0.79	5.3

Table S3 The Brunauer-Emmet-Teller (BET) surface area and pore volume of the obtained catalysts.

	Co_3S_4-S/G-600	Co_3S_4-S/G-700	Co_3S_4-S/G-800	Co_3S_4-S/G-900
**BET surface area (m^2 g^{-1})**	26.24	52.02	52.44	38.36
**Pore volume (cm^3 g^{-1})**	0.084	0.106	0.121	0.118
**Average pore size (nm)**	11.2	8.5	13	10.4

Table S4 Comparison of the ORR ability of various catalysts.

Catalysts	Fabrication method	Electrolyte	Potential at J=3mA cm^{-2} (V) vs. RHE	Electron transfer number (n)	Reference
Co_3S_4-S/G-800	Pyrolysis a S_4-chelate complex and GO	0.1 M KOH<br>0.5 M H_2SO_4	0.792<br>0.42	3.88~4<br>3.83~3.93	This work
$Co_{1-x}S$/RGO hybrid	Two steps: solution-phase reaction process and a annealing process	0.1 M KOH<br>0.5 M H_2SO_4	0.75<br>0.42	4<br>3.4~3.8	1
Co_3S_4/Graphene Composites	Multi-step hydrothermal reaction process	0.1 M KOH<br>0.5 M H_2SO_4	0.53<br>0.11	3.2~3.9<br>2.0~3.2	2
CoS_2-based thin films	magnetron sputtering	0.1 M $HClO_4$	0.34	—	3
CoS_2/N,S-GO	Pyrolysis of cobalt thiourea and GO	0.1 M KOH	0.79	3.81	4
$Co_{0.5}Fe_{0.5}S$@N-MC	Pyrolysis of thiourea and pluronic F127 in presence of cobalt(II) acetate and iron(III) nitrate	0.1 M KOH	0.79	3.8~4	5
Fe-P-C	Pyrolysis of phytic acid in presence of ferric trichloride	0.1 M KOH<br>0.1 M $HClO_4$	0.75<br>0.45	3.83<br>3.61	6
g-C_3N_4/C	By a hard template method fabricating graphitic carbon nitirde	0.1 M KOH	0.6	3.17	7

—: no data presented.

References

1. Wang, H. L.; Liang, Y. Y.; Li, Y. G.; Dai, H. J. *Angew. Chem. Int. Ed.* **2011**, *50*, 10969.
2. Mahmood, N.; Zhang, C. Z.; Jiang, J.; Liu, F.; Hou, Y. L. *Chem. Eur. J.* **2013**, *19*, 5183.
3. Zhu, L.; Susac, D.; Teo, M.; Wong, K. C.; Wong, P. C.; Parsons, R. R.; Bizzotto, D.; Mitchell, K. A. R.; Campbell, S. A. *J. Catal.* **2008**, *258*, 235.
4. Sun, T.; Wu, Q.; Che, R. C.; Bu, Y. F.; Jiang, Y. F.; Li, Y.; Yang, L. J.; Wang, X. Z.; Hu, Z. *ACS Catal.* **2015**, *5*, 1857.
5. Shen, M. X.; Ruan, C. P.; Chen, Y.; Jiang, C. H.; Ai, K. L.; Lu, L. H. *ACS Appl. Mater. Interfaces* **2015**, *7*, 1207.
6. Singh, K. P.; Bae, E. J.; Yu, J. S. *J. Am. Chem. Soc.* **2015**, *137*, 3165.
7. Liang, J.; Zheng, Y.; Chen, J.; Liu, J.; Jurcakova, D. H.; Jaroniec, M.; Qiao, S. Z. *Angew. Chem. Int. Ed.* **2012,** *51*, 3892.
